# Assessing predictors for new post translational modification sites: A case study on hydroxylation

**DOI:** 10.1371/journal.pcbi.1007967

**Published:** 2020-06-22

**Authors:** Damiano Piovesan, Andras Hatos, Giovanni Minervini, Federica Quaglia, Alexander Miguel Monzon, Silvio C. E. Tosatto

**Affiliations:** Department of Biomedical Sciences, University of Padua, Padua, Italy; University of California San Diego, UNITED STATES

## Abstract

Post-translational modification (PTM) sites have become popular for predictor development. However, with the exception of phosphorylation and a handful of other examples, PTMs suffer from a limited number of available training examples and sparsity in protein sequences. Here, proline hydroxylation is taken as an example to compare different methods and evaluate their performance on new experimentally determined sites. As a guide for effective experimental design, predictors require both high specificity and sensitivity. However, the self-reported performance may often not be indicative of prediction quality and detection of new sites is not guaranteed. We have benchmarked seven published hydroxylation site predictors on two newly constructed independent datasets. The self-reported performance is found to widely overestimate the real accuracy measured on independent datasets. No predictor performs better than random on new examples, indicating the refined models do not sufficiently generalize to detect new sites. The number of false positives is high and precision low, in particular for non-collagen proteins whose motifs are not conserved. As hydroxylation site predictors do not generalize for new data, caution is advised when using PTM predictors in the absence of independent evaluations, in particular for highly specific sites involved in signalling.

## Author summary

Machine learning methods are extensively used by biologists to design and interpret experiments. Predictors which take the only sequence as input are of particular interest due to the large amount of available sequence data and high self-reported performance. In this work, we evaluated post-translational modification (PTM) predictors for hydroxylation sites and found that they perform no better than random, in strong contrast to performances reported in their original publications. PTMs are chemical amino acid alterations providing the cell with conditional mechanisms to fine tune protein function, regulating complex biological processes such as signalling and cell cycle. Hydroxylation sites are a good PTM test case due to the availability of a range of predictors and an abundance of newly experimentally detected modification sites. Poor performances in our results highlight the overlooked problem of predicting PTMs when best practices are not followed and training data are likely incomplete. Experimentalists should be careful when using PTM predictors blindly and more independent assessments are needed to establish their usefulness in practice.

This is a *PLOS Computational Biology* Benchmarking paper.

## Introduction

Post translational modifications (PTMs) are alterations of the primary protein structure, including both new covalent links and cleavage events. Almost every protein in the cell undergoes modification during its lifetime [1] and more than 600 different amino acid modifications are catalogued in UniProtKB [2]. PTMs provide a way to expand the spectrum of protein functions as well as an additional layer for pathway regulation [3]. They are catalyzed by enzymes that identify a specific site in the substrate protein, with a plurality of PTM motifs residing in intrinsically disordered regions in order to facilitate enzyme accessibility [4]. Over the last few years, a deluge of methods have been proposed to predict PTM sites from sequence, for a recent review see e.g. [5]. The reasons for this popularity are broadly twofold. Given the paucity of experimental data for PTMs and their relevance for cellular regulation, there is a legitimate expectation that computational methods should fill in the experimental void. Computational methods can become hypothesis generators for an effective design of PTM experiments. Their implementation is straightforward due to the sequence specificity and peculiar physico-chemical properties of PTM motifs. This simplicity makes PTM prediction from sequence easily accessible to machine learning methods, but also presents several potential pitfalls [6]. In order to be useful for experimentalists, PTM predictors should provide good performance and be robust. Performance should be high enough to limit false positives to a minimum, while ensuring sufficient amount of correct predictions (true positives). Perhaps more importantly, the method should be robust enough to maintain performance across a range of different datasets, as it is often not clear which experimental conditions may introduce biases. On both accounts, PTM predictors may be problematic as they are rarely assessed by independent third parties. Indeed, their ability to identify new modification sites has been questioned [7] and effective results have been obtained only for a few PTM types [5]. The problem of validating machine learning methods has already been raised and best practices have been proposed [6]. Self-reported accuracy may be overestimated, with PTM predictors overfitting and not performing better than random when adopting the wrong training strategy [7]. Generalizing models for PTM site recognition is difficult as the number of experimental observations is low and many new types of motifs are still poorly characterized.

In this work, proline hydroxylation is taken as a case study to answer the question of how useful PTM predictors, especially those trained on small datasets, are to design experiments. Hydroxylation is one of the most abundant PTMs in the cell [8]. However, despite improvements in mass-spectrometry (MS) techniques, likely only a small fraction of all hydroxylated sites has so far been experimentally detected.

Proline hydroxylation (PH) is a PTM carried out by prolyl hydroxylases, catalyzing the addition of a hydroxyl group to the sidechain pyrrolidine ring at the gamma position. This modification is crucial for correct folding of the collagen triple-helix, which contains the conserved xPG motif. PH also plays a crucial role in signaling, in particular in oxygen sensing pathways, angiogenesis [9] and tumor cell proliferation [10, 11]. An example is HIF1α, the main target of the von Hippel-Lindau (pVHL) E3 ubiquitin ligase complex [12]. In normoxia, the prolyl hydroxylase domain-containing enzymes (PHDs) hydroxylate HIF1α, promoting its degradation through pVHL binding [13]. Under low oxygen concentration, the PHDs are inactivated and HIF-1α translocates into the nucleus to activate vascular proliferation and angiogenesis genes [14].

The first hydroxylation predictor [15] was trained to predict only collagen modifications. Several further PH predictors exist as web servers: HydPred [16], PredHydroxy [17], RF-Hydroxysite [18], iHyd-PseAAC [19] and iHyd-PseCp [20]. The latter has not been considered in our analysis as the server proved unstable, with frequent freezes. The stand-alone PH software OH-Pred [21], ModPred [4] and AMS3 [1] are also available. All are potential tools for large-scale analysis, taking only the protein sequence as input. Implementations include standard machine learning algorithms like Support Vector Machines, artificial Neural Networks and Random Forests, as well as alternative techniques like logistic regression and probabilistic classifiers. All methods were trained on SwissProt [22] annotation, with varying strategies to define positive and negative examples and different approaches to evaluate model quality. None of the PH predictors used a real independent dataset for validation, i.e. unaffected from SwissProt biases.

Here, we evaluate PH methods considering separately collagen and signalling examples as well as single proteins versus high throughput mass-spectrometry (MS) experiments. The majority of new hydroxylated prolines (Hyp) come from two MS recently published experiments, one on HeLa cells and another from a large experiment involving multiple tissues and samples [23–25]. These datasets are unseen for the PH predictors being tested, as they were not yet available in public databases when the predictors were trained. The number of MS hydroxylated sites is comparable to the entire SwissProt database and the new datasets allowed us to perform an unbiased blind test. A Naïve HMM predictor trained including MS data has also been implemented to simulate the effect of integrating new examples. The analysis presented here provides a starting point for a critical discussion on the problem of reliably predicting new PTMs.

## Results

The intended users of PTM predictors are experimentalists working intending to make better use of their limited time and budget. While prediction tools have the potential to make experiments more effective, they need to work much better than random. In the case of PTMs, where the fraction of modified residues is low, the false positive rate (i.e. fraction of false positives among predictions made) should be minimized. Due to the low coverage of PTM evidence in public databases, predictors also need to generalize well, i.e. identify new motifs never seen before. In the following, different PH predictors are evaluated against new hydroxylation sites and various problems related to PTM prediction are discussed. In order to provide an objective evaluation we considered “old” examples from the SwissProt database at the time of PH method training (Literature) and “new” examples coming from two different mass-spectrometry experiments (MS-Kim, MS-HeLa). Since collagen is a recurrent PH substrate with well-defined sequence pattern, “new but easy” examples (MS-collagen) are a subset of the “new” examples found in collagen proteins similar to the “old” collagen motifs used for training PH methods. Table 1 shows the main splits used in this paper. For methods which provide a confidence score, the Precision-recall and ROC curves are reported in Fig C-H in S1 Text.

**Table 1:** Datasets. Negative clusters (“Negative”) contain only clusters with non-hydroxylated sites. Other datasets have clusters with both positive and negative examples, but negatives are completely removed during evaluation (*). Negative sites (^) considered during evaluation are always resampled for each replica, based on the size of the positive dataset.

Dataset	Clusters	Evaluated sites	Filtered negative sites*
Collagen	5	243	4,517
Literature-collagen	4	152	4,588
MS-collagen	4	95	4,508
Literature	167	877	13,481
MS-HeLa	198	324	14,982
MS-Kim	625	1,694	24,692
MS	705	2,002	26,631
Negative	493	7,875^	-

### Predictor performance

**Fig 1:**
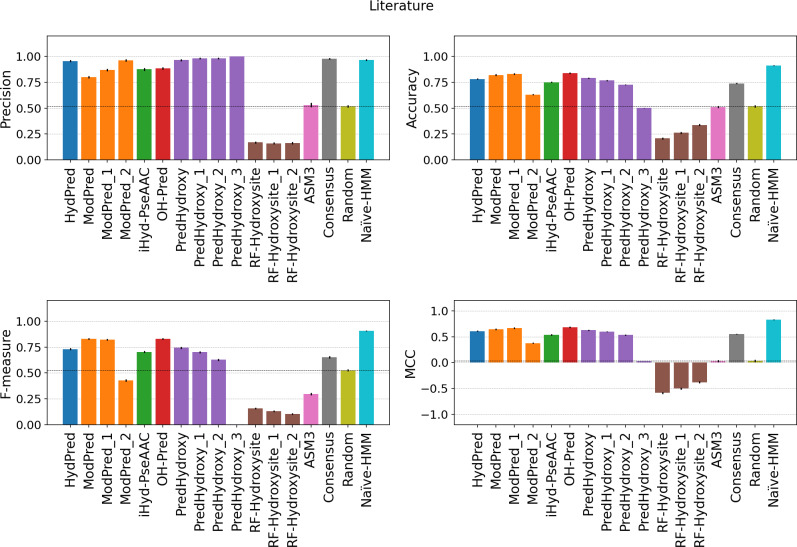
Performance on literature examples. The evaluation is performed only considering hydroxylated sites detected by single protein experiments (Literature dataset). Error bars are the standard deviation calculated over 1,000 replica sets. The consensus baseline method is the majority vote across all predictors. Suffix numbers in the method names indicate increasing quality threshold as defined by developers.

As a starting point, Table 2 shows details about the evaluated predictors, including self-reported performance. Self-reported performance is taken from the corresponding publications selecting values calculated on independent validation sets where possible, i.e. excluding training examples. The performance considering manually curated examples from single protein experiments (Literature) is shown in Fig 1 to simulate the evaluation provided by the method publications. The majority of “Literature” examples in fact come from SwissProt and were already available at the training time. While ModPred, HydPred and OH-Pred perform as declared, ASM3, PredHydroxy and RF-Hydroxysite all show a decrease. The RF-Hydroxysite performance is worse than random with a negative MCC, probably because its web server suffers from a software bug. For the best methods, sensitivity and specificity are both high (Table A in S1 Text). Methods providing a confidence threshold can modulate the precision (PPV) correctly, with the exception of PredHydroxy which at high confidence (0.9) has a sensitivity of zero and behaves like a random predictor.

**Table 2:** Methods overview. Self-reported performance is taken from the corresponding method publications preferring values reported from independent validation sets, i.e. not used in the training. The “Type” column indicates the type of hydroxylated residue predicted, proline (P), lysine (K) and tyrosine (Y). “Window” indicates the number or neighbour residues considered for a prediction. Self-reported performance includes specificity (Sp), sensitivity (Sn), accuracy (acc), Matthew’s Correlation Coefficient (MCC) and the area under the ROC curve (AUC).

Method	Implementation	Availability	Training set available	Type	Window	Self-reported performance				
						Sp	Sn	Acc	MCC	AUC
**AMS3** *Basu et al*. *2010*	Neural network	Stand alone	no	P,K	9	-	0.95	-	-	0.97
**HydPred** *Li et al*. *2016*	Random forest	Web service	yes	P,K	13	0.89	0.71	0.85	0.60	-
**iHyd-PseAAC** *Xu et al*. *2014*	Vector similarity	Web service	yes	P,K	13	0.79	0.71	0.75	0.52	-
**ModPred** *Pejaver et al*. *2014*	Logistic regression	Stand alone	yes	P,K,Y	21	0.90	0.54	0.72	-	0.83
**OH-Pred** *Shi et al*. *2015*	Support Vector Machine	Stand alone	no	P,K	15	0.82	0.76	0.81	0.52	-
**PredHydroxy** *Jia et al*. *2017*	Support Vector Machine	Web service	yes	P,K	13	0.87	0.96	0.92	0.83	-
**RF-Hydroxysite** *Ismail et al*. *2016*	Random forest	Web service	no	P,K	13	0.96	0.97	0.96	0.93	-

**Fig 2:**
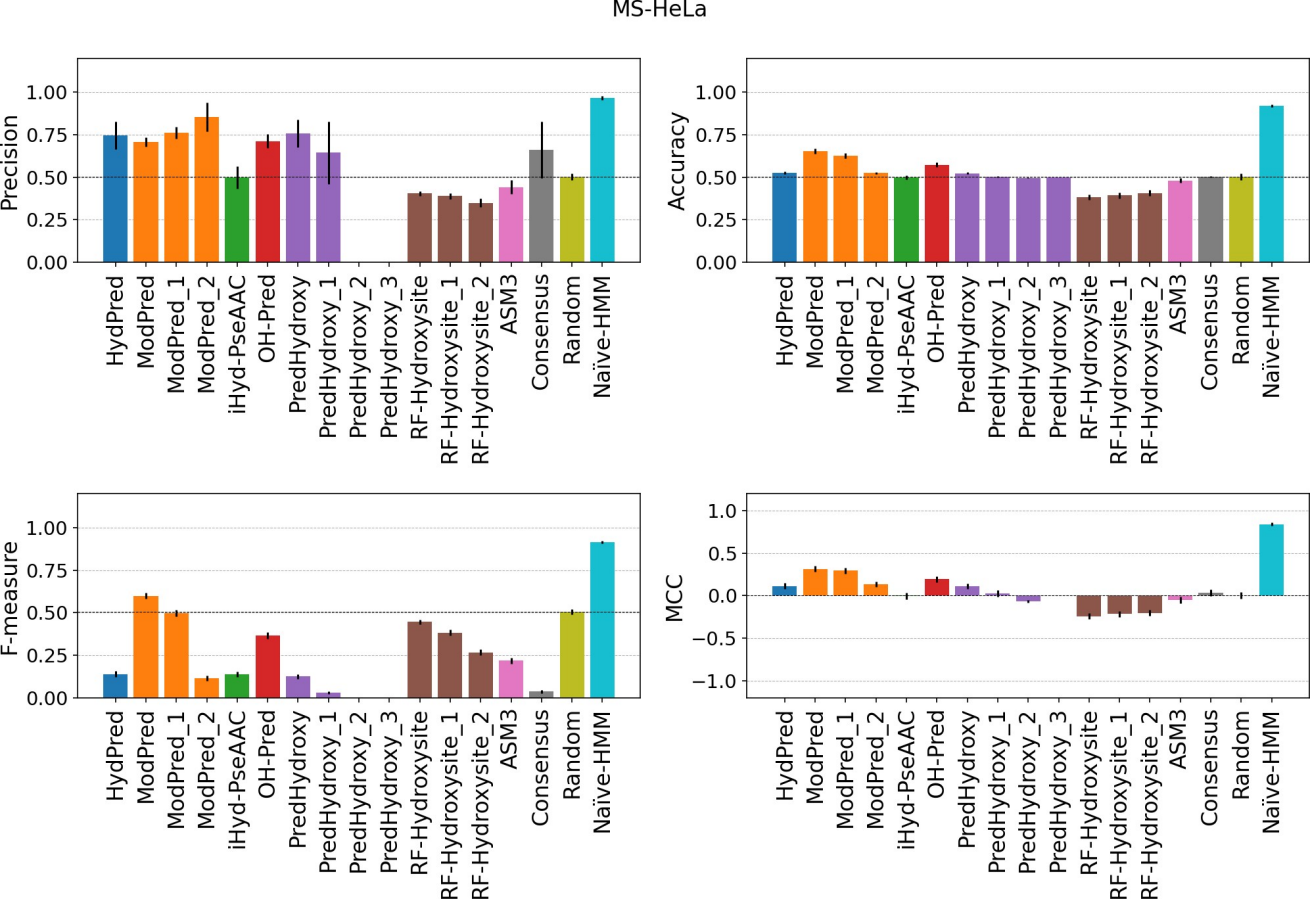
Performance on the MS-HeLa datasets. The evaluation is performed only considering hydroxylated sites detected by a mass-spectrometry experiment (MS-HeLa dataset). Consensus and errors are calculated as in the previous figure. Suffix numbers in the method names indicate increasing quality threshold as defined by developers.

**Fig 3:**
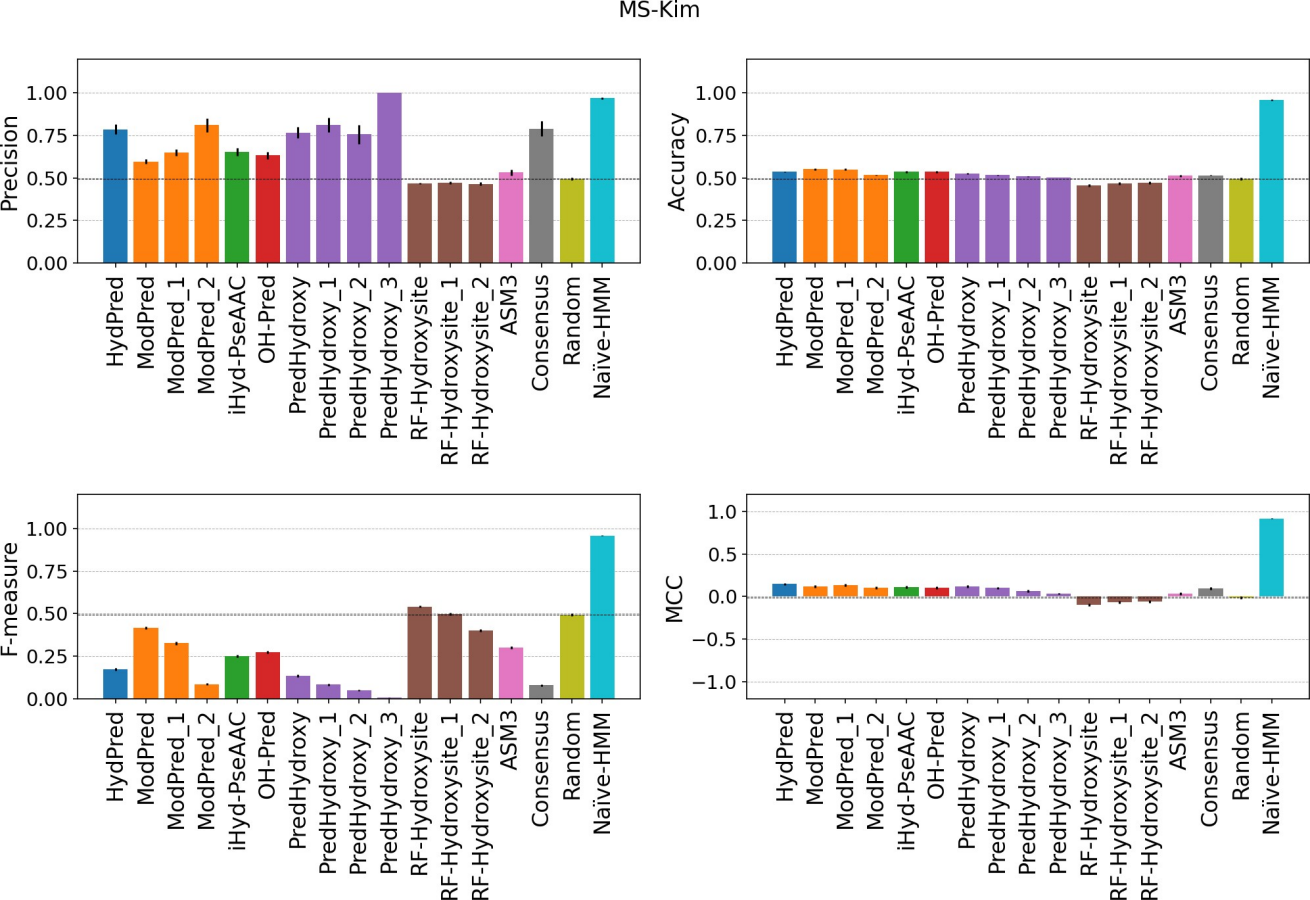
Performance on the MS-Kim datasets. The evaluation is performed only considering hydroxylated sites detected by a mass-spectrometry experiment (MS-Kim dataset). Consensus and errors are calculated as in the previous figure. Suffix numbers in the method names indicate increasing quality threshold as defined by developers.

All methods do not seem to generalize well and have low sensitivity on the new MS examples. Even if there are substantial differences in the MS site detection protocol between the MS-HeLa and MS-Kim datasets, predictor behavior is very similar (Figs 2 and 3 and Tables B-C in S1 Text). Both absolute values and predictor rankings change significantly when measuring the performance on new MS examples (Table D in S1 Text). High specificity combined with low sensitivity, e.g. PredHydroxy, is critical in particular for unbalanced and incomplete datasets like PTMs. In this context, it simply means the predictor is classifying the majority of sites as negatives. Since the positive to negative ratio for PH in the human genome is less than 10%, negative examples might become positive as new experimental evidence is collected. This would result in an increased false negative rate and decreased sensitivity. Such a behaviour is typical of overfitted models trained on biased datasets, which are unable to generalize. All predictors have a balanced accuracy close to 0.5, indicating a random behavior. Notably, only ModPred is better than random for the MS-HeLa dataset (Fig 2 and Table B in S1 Text), achieving the highest MCC, 0.13 in MS-Kim (Fig 3) and 0.32 in MS-HeLa (Fig 2), which is still not sufficient for practical use by experimentalists. For example, considering the merged MS dataset, only 62% of positive hydroxylation site predictions will be correct (precision) and 65% of modified residues undetected (false negative rate) (Table D in S1 Text). We explored the possibility of reducing false positives by implementing a consensus predictor based on a majority vote. In all evaluations the consensus is in line with the method average. This again highlights how methods are unable to generalize, with predictors agreeing only on a very small subset of positive sites, shown by the low sensitivity in the MS dataset. Overall, it is fair to say that the predictors do not work well on the new datasets. The NaÏve-HMM baseline (see Materials & Methods), is trained as an upper estimate for predictor performance using also new examples. Since its training set overlaps the benchmarking set it behaves like a perfect predictor. However, while its ability to generalize is not proven, it demonstrates how negative sites are significantly different from positives and predictors can benefit from incorporating new sites in training.

### Collagen

**Fig 4:**
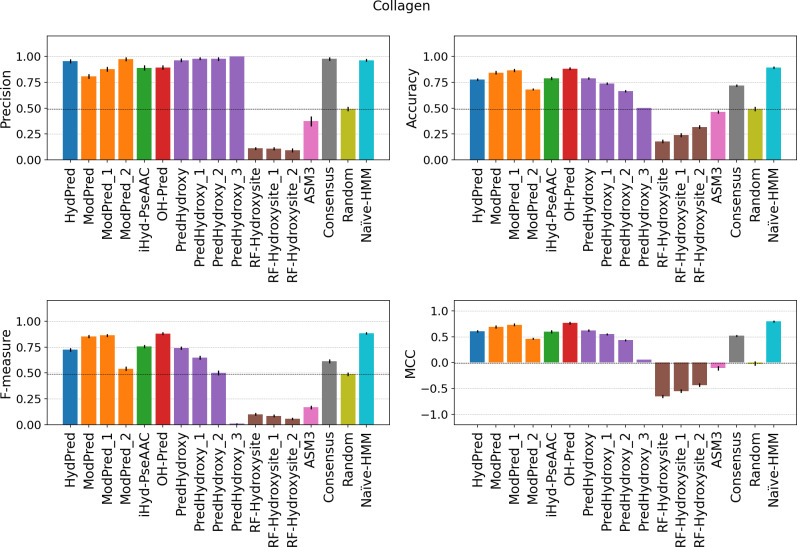
Performance on MS-collagen examples. The evaluation is performed only considering hydroxylated sites detected by mass-spectrometry experiments and belonging to collagen proteins (MS-collagen dataset). Consensus and errors are calculated as in the previous figure. Suffix numbers in the method names indicate increasing quality threshold as defined by developers.

A case of special interest may be collagen, which accounts for very specific hydroxylation motifs. In collagen, hydroxylation affects different locations corresponding to different sites and molecular meanings. Collagen presents the canonical Xaa-Yaa-Gly pattern with the Pro-Hyp-Gly triplet found in 10.5% of collagen motifs [26]. Xaa is a Proline in 28% and Yaa is Hyp in 38% of the cases. The Hyp position matters, as in Xaa it prevents the formation of the tropocollagen (TC) triple helix [27]. Collagen motifs are conserved, well studied and easier to predict compared to signaling hydroxylation. Both the Literature and MS datasets include 152 and 95 collagen sites respectively (Table 1), all grouped into only 5 different clusters (Fig B in S1 Text). Unsurprisingly, considering Literature collagen motifs, predictors stand out achieving a maximum MCC of 0.81 and accuracy of 0.94 (Table C in S1 Text). The situation is similar when considering collagen examples from the MS dataset (Fig 4), indicating the quality of the MS data is comparable to the Literature data. Comparing these results with the MS dataset performance, it can be concluded that methods were trained for predicting collagen sites rather than hydroxylation sites in general. With the exception of ModPred, the low sensitivity on the MS collagen dataset indicates methods were not trained following the best practices, resulting in an overfitting of the predictors.

### Dataset characterization

**Fig 5:**
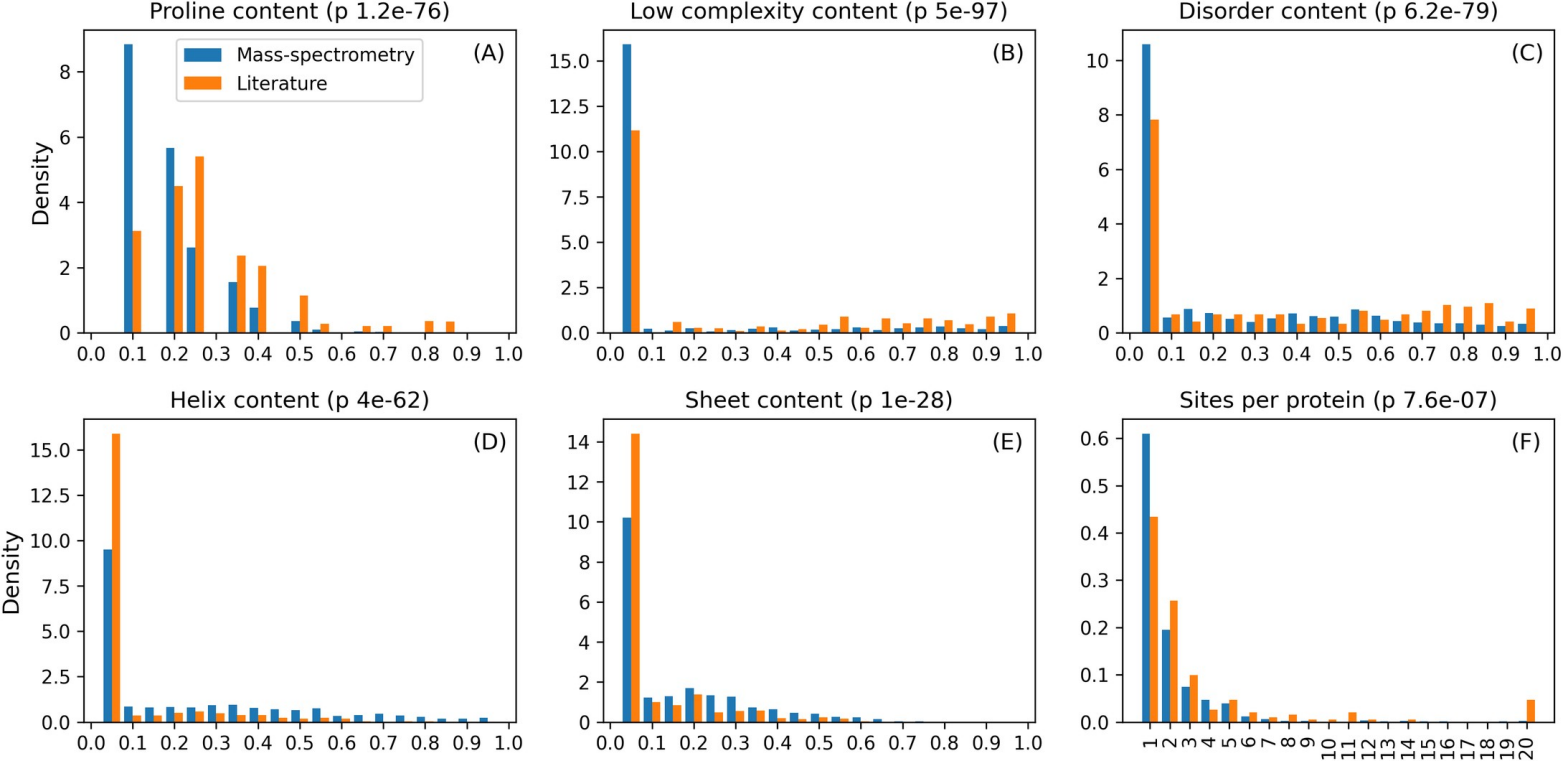
Features distribution for MS and Literature sites. Content refers to the fraction of residues in the site sequence associated with a given feature. Density refers to the fraction of proteins in the dataset with a given number of sites.

Given the difference in performance across datasets, we tried to characterize better potential differences in the datasets. Hydroxylation is known to be linked to angiogenesis and tumor growth and hydroxylases have been observed to be particularly active [28] with changing collagen patterns in cancer cells [29]. Therefore, we distinguished between examples from single-protein experiments described in the literature and hydroxylation observed in tumor cells from MS experiments. MS experiments are not free from bias [30] and enriched in flexible peptides [31]. One of the two MS experiments has also been performed using an anti-hydroxyproline antibody which may induce a sequence bias. The analysis was therefore limited to high confidence sites with at least 80% experimental score probability. Considering sequence site similarity, the Literature dataset is a subset of the MS dataset. 122 out of 165 Literature clusters (74%) have at least one MS site (intersection). These clusters include 92% of the total Literature examples (13,196 sites). On the other hand, 583 clusters including 15,437 sites have only MS examples representing the real new hydroxylation motifs. In order to assess non-specific proline binding, a comparison between the MS and Literature datasets is reported in Fig 5. The residue frequencies around the modified proline (Panel A) decay exponentially for the MS dataset while Literature sites have a peak at 25%, with a distribution shifted towards enriched sites. This is probably due to a stronger contribution of highly repeated collagen patterns. A supposed bias towards polyproline detection by MS experiments is however not observed. The number of hydroxylated sites per protein (Fig 5F), despite being similar for the two datasets, shows more sites per protein in Literature which might also be related to collagen abundance. The expected over-hydroxylation of MS sites is again not observed. Three quarters of the sites are hydroxylated 100% of the time according to the MS results, but we do not have this information for the Literature dataset. As previously observed for several PTM types [4], hydroxylation also has a preference for intrinsically disordered regions (Fig 5C) rather than for secondary structure elements (Fig 5D and 5E) or low complexity regions (Fig 5B). In summary, even if the distributions are statistically different, no particular evidence was found for a specific sequence-based bias in the MS dataset compared to Literature examples. This suggests that the predictors, if properly trained, should be able to generalize sufficiently to predict MS hydroxylation sites.

## Discussion

We assessed hydroxylation site predictors as a paradigm for common situations arising with sequence-based machine learning methods [6]. Our analysis using unbiased testing data set suggests that predictors perform no better than random when predicting hydroxylation PTM on new examples, which make them unsuitable for experimental biologists. This is in strong contrast to the self-reported performances on independent datasets. Bad performance on new examples can be explained by one of two reasons. First, a bad training protocol and second, an intrinsic problem of machine learning methods able to detect only patterns highly similar to training examples. For the hydroxylation predictors assessed here, problems in the training protocol also extend to the construction of the dataset. Some methods choose negatives from complementary sites in hydroxylated sequences, while others randomly select negatives from non-hydroxylated sequences. The first strategy might be more reliable, since presumably both positive and negative sites have been tested experimentally. On the contrary, randomly selected proteins might include modifications not observed yet. Some methods generate training sets by filtering negative sites, using solvent accessibility predictions to exclude positions on the protein surface. This is problematic since it can introduce additional uncertainty when surface residues are mispredicted. Another critical point is the sequence redundancy in the training set. All methods, with the exception of ModPred, reduce redundancy at the protein level. This is problematic since protein pairs with low global identity can share short regions with high similarity including the hydroxylation sites. Even more problematic is the choice of the validation set. When both the training and validation sets include the same bias, predictors will over-weight biased features and perform poorly on new examples. Besides technical problems related to machine learning, predicting PTMs is particularly difficult as different modification patterns can be observed in different cells as well as in response to environmental conditions or disease states. Hydroxylation apparently does not escape this paradigm and predictors are not able to provide novel hypotheses. While new data generated by MS experiments will improve predictor accuracy and sensitivity, at the moment it is hard to estimate the amount of examples necessary to represent the entire PTM motif space. This is particularly critical for PTMs in general as they are heavily involved in the regulation of biological processes/signaling and have an extremely dynamic turnover.

In conclusion, we have provided a thorough independent assessment of previously published hydroxylation site predictors. Our results do not bode well for the field, suggesting that self-reported performance is often overestimated and difficult to replicate. This should be seen as an example for the common pitfalls associated with many of the current PTM predictors. Knowing how well training sets cover the real PTM distribution is crucial. Experimentalists should be careful when using PTM predictors until more independent assessments are able to establish the true state-of-the-art.

## Materials and methods

### Dataset

Hydroxylated substrate sequences were retrieved from SwissProt [32] (version 2018_03) considering all organisms. The dataset is further filtered by retaining only manually curated annotations with evidence code “experimental evidence used in manual assertion” (ECO:0000269) or “curator inference used in manual assertion” (ECO:0000305). UniProtKB provides a controlled vocabulary of all PTM types, of which the following terms are considered for our PH analysis: 4-hydroxyproline (1,033 sites), hydroxyproline (220 sites), 3-hydroxyproline (27 sites), 3,4-dihydroxyproline (5 sites) and (3R,4R)-3,4-dihydroxyproline (1 site). Additional PH sites are retrieved from the literature [33–36] including two large scale MS experiments, one on HeLa cells [23] and another based on 30 normal human samples including almost all tissues [24], reanalysed with a new software, TagGraph (PRIDE accession PXD005912) [25]. MS experiments provide the majority of new examples currently not included in SwissProt. HeLa examples are filtered to retain only sites with a confidence probability of 0.8 to minimize assignment errors. Compared to the original analysis [24], TagGraph on average tripled the number of identified sites with a degree of variability depending on the tissue type [25]. Even though some PH methods predict lysine and tyrosine hydroxylation, only prolines are considered in the assessment in order to reach statistical significance given the paucity of data for other PTMs. All predictors identify modified residues exploiting the sequence context (surrounding residues) with the assumption that it encodes sufficient information for molecular recognition. The maximum window size adopted by the methods used in this study is 21 residues.

**Fig 6:**
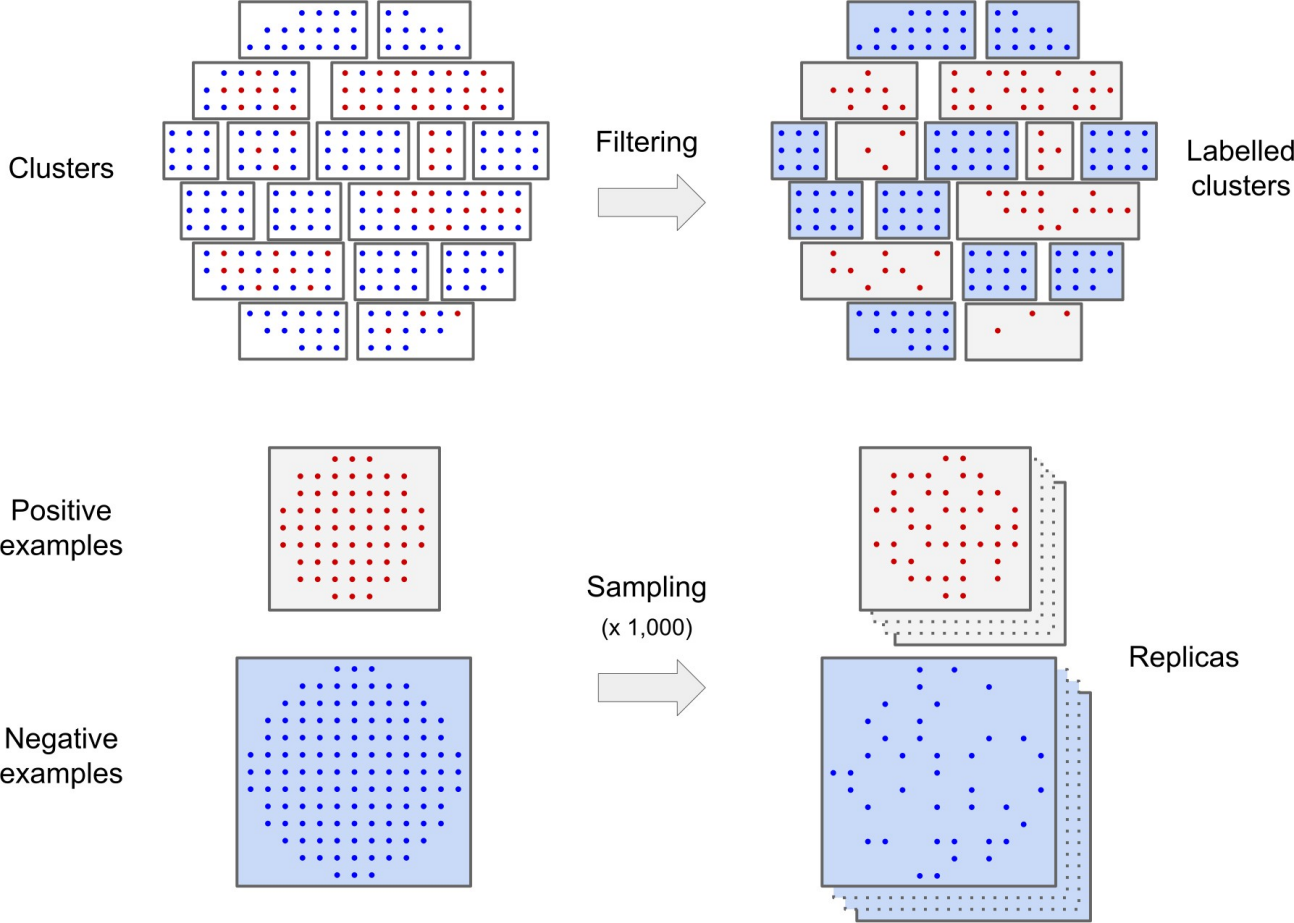
Dataset generation. Negative (blue dots) and positive sites (red dots) are clustered based on sequence similarity. Positive clusters (gray background) contain at least one hydroxylation site and negative examples falling inside positive clusters are removed. 1,000 replica sets are created by random sampling 70% of the positive sites and the same number from the negatives.

The final dataset includes 1,419 proteins with at least one hydroxylated proline and output for all predictors. Only 10% of all prolines are hydroxylated (3,771 out of 37,670). Sites are defined considering a window of 13 residues centered on the proline. The evaluation of predictors has been performed on a subset of sites selected as follows (Fig 6). Both positive and negative sites are clustered together based on sequence similarity using a distance matrix representing the pairwise sequence divergence between all sites. The distance is computed as the inverse of the similarity score, which is calculated elementwise for each pair of residues of the two sequence sites. The score of a single pair is taken from the Blosum62 matrix, with a penalty of -5 for gap opening and -1 for gap extension. Gaps are introduced to substitute non-canonical residues or to pad the site sequence to reach the window size when a proline is too close to sequence ends. Fig A in S1 Text shows the dendrogram of hierarchical clustering calculated with the UPGMA algorithm implemented in the SciPy library. Negative examples too similar to positive examples are removed, i.e. when falling inside clusters containing at least one hydroxylated proline (positive site). The evaluation of the predictors is performed on 1,000 different balanced replica sets, each built by random picking 70% of the available positive sites and the same number of negatives.

The evaluation is provided for different subsets of positive examples. Namely, sites from single protein experiments (Literature) and mass-spectrometry (MS-Kim, MS-HeLa). The two mass-spectrometry datasets were also merged for some of the presented analysis (MS dataset). Both the Literature and MS datasets can be further divided into collagen and non-collagen entries by recognizing the collagen motif from Pfam domain annotation PF01391 [37]. The corresponding collagen site datasets are respectively called Literature-collagen and MS-collagen. The tested examples are resampled as described above for each subset and each replica. To further characterize the dataset sequences, secondary structure (helix/sheet propensity) is predicted using FELLS [38], functional disorder with MobiDB-lite [39] and low complexity with SEG [40]. All predictors were executed on full protein sequences. The fraction of residues assigned to a given feature (content) is calculated for each site. Proline content is calculated as the fraction of prolines in the site irrespective of any hydroxylation modification.

### Prediction

We evaluated seven different PH predictors on entire protein sequences, implemented as either stand-alone software or web server. Since no web server allowed for programmatic access, all predictions were parsed from web page results. The iHydPse-CP web server [20] stopped working during the benchmark and was excluded from the evaluation as it was never restored. Another method is described in the literature [41] but the software has not been released even upon request. Some methods are designed to predict different modification types. Our evaluation focuses only on proline hydroxylation in order to provide statistically significant results. Some predictors (HydPred, ModPred, PredHydroxy, RF-Hydroxysite) estimate prediction quality providing a confidence value. When different quality levels are provided, we evaluated them as different predictors. In all figures, suffix numbers in method names indicate increasing quality threshold as defined by the developers. The random baseline method predicts each site randomly as hydroxylated with 50% probability. We decided not to include a separate random baseline with a probability proportional to the data imbalance, since this probability is difficult to estimate for hydroxylation. The random baseline represents a situation where prediction is effectively useless and predictors should achieve significantly better results to be of any practical value for experimentalists.

### Naive HMM

The “Naive-HMM” baseline method has been implemented to demonstrate that negative examples are very different from positive sites and that they may be correctly classified by integrating new information into training datasets. A database of 750 HMMs representing hydroxylated motifs were built considering those clusters containing at least one positive site as seeds using the HMMER software [42]. Naive-HMM predictions were generated by aligning all dataset sites against the HMM database. Hits with an alignment E-value better than 1.0 are considered positive predictions. The very permissive E-value is necessary as sequence sites are very short compared to full Pfam domains. Less permissive E-value thresholds do not significantly affect the performance. It should be noted that positive examples in the training and test sets overlap completely, even if negative sites inside HMM seeds are retained. Notice that the Naive-HMM baseline is not meant to be of any use for biologists and does not guarantee to generalize for new sites, rather it is intended as an upper limit for predictor performance.

### Evaluation

The assessment is site centric, i.e. all modified (and non-modified) prolines are considered independent examples when belonging to the same protein. True positives (TP) correspond to correctly predicted hydroxylation sites, whereas false positives (FP) are prolines predicted as modified in contradiction to experimental observations. True negatives (TN) are sites predicted and observed as not hydroxylated and false negatives (FN) are negative predictions of truly modified prolines. Sensitivity (Sn), specificity (Sp), weighted (or balanced) accuracy (WACC), F-measure (F1), Precision or Positive Predictive Value (PREC) and Matthew’s correlation coefficient (MCC) are computed using standard definitions. Even where not mentioned explicitly, accuracy is always balanced.

## Supporting information

S1 Text Supplementary tables and figures. (DOCX)

S1 Data Evaluation source code, predictions and reference datasets. (ZIP)
