# Supplementary material for: Assessing predictors for new post translational modification sites: A case study on hydroxylation
Source: PLoS Comput Biol. 2020 Jun 22;16(6):e1007967. doi: 10.1371/journal.pcbi.1007967 (PMC7332089; doi:10.1371/journal.pcbi.1007967)
Supplement: S1 Text — (DOCX) [file pcbi.1007967.s001.docx]

**Assessing predictors for new post translational modification sites: a case study on hydroxylation**

Damiano Piovesan^1,*^, Andras Hatos^1^, Giovanni Minervini^1^, Federica Quaglia^1^, Alexander Miguel Monzon^1^ and Silvio C.E. Tosatto^1^

^1^ Department of Biomedical Sciences, University of Padua, Padua, Italy.

* damiano.piovesan@unipd.it

**Supplementary tables and figures**

| **Method** | **Sn** | **Sp** | **Prec** | **Wacc** | **F1** | **Mcc** |
| --- | --- | --- | --- | --- | --- | --- |
| HydPred | 0.59 | 0.97 | 0.96 | 0.78 | 0.73 | 0.61 |
| ModPred | 0.86 | 0.78 | 0.80 | 0.82 | 0.83 | 0.64 |
| ModPred_1 | 0.78 | 0.88 | 0.87 | 0.83 | 0.82 | 0.67 |
| ModPred_2 | 0.27 | 0.99 | 0.96 | 0.63 | 0.43 | 0.38 |
| iHyd-PseAAC | 0.59 | 0.92 | 0.88 | 0.75 | 0.70 | 0.53 |
| OH-Pred | 0.78 | 0.90 | 0.88 | 0.84 | 0.83 | 0.68 |
| PredHydroxy | 0.60 | 0.98 | 0.96 | 0.79 | 0.74 | 0.63 |
| PredHydroxy_1 | 0.55 | 0.99 | 0.98 | 0.77 | 0.70 | 0.60 |
| PredHydroxy_2 | 0.46 | 0.99 | 0.98 | 0.73 | 0.63 | 0.53 |
| PredHydroxy_3 | 0.00 | 1.00 | 1.00 | 0.50 | 0.00 | 0.03 |
| RF-Hydroxysite | 0.15 | 0.27 | 0.17 | 0.21 | 0.16 | -0.59 |
| RF-Hydroxysite_1 | 0.11 | 0.41 | 0.16 | 0.26 | 0.13 | -0.50 |
| RF-Hydroxysite_2 | 0.08 | 0.60 | 0.16 | 0.34 | 0.10 | -0.38 |
| ASM3 | 0.21 | 0.82 | 0.53 | 0.51 | 0.30 | 0.03 |
| Consensus | 0.49 | 0.99 | 0.98 | 0.74 | 0.65 | 0.55 |
| Random | 0.54 | 0.50 | 0.52 | 0.52 | 0.53 | 0.03 |
| Naïve-HMM | 0.86 | 0.97 | 0.97 | 0.91 | 0.91 | 0.83 |

**Table A. Performance on the Literature dataset.**

| **Method** | **Sn** | **Sp** | **Prec** | **Wacc** | **F1** | **Mcc** |
| --- | --- | --- | --- | --- | --- | --- |
| HydPred | 0.08 | 0.97 | 0.75 | 0.52 | 0.14 | 0.11 |
| ModPred | 0.52 | 0.78 | 0.71 | 0.65 | 0.60 | **0.32** |
| ModPred_1 | 0.37 | 0.88 | 0.76 | 0.63 | 0.50 | 0.29 |
| ModPred_2 | 0.06 | 0.99 | 0.85 | 0.52 | 0.11 | 0.13 |
| iHyd-PseAAC | 0.08 | 0.92 | 0.50 | 0.50 | 0.14 | 0.00 |
| OH-Pred | 0.25 | 0.90 | 0.71 | 0.57 | 0.37 | 0.19 |
| PredHydroxy | 0.07 | 0.98 | 0.76 | 0.52 | 0.12 | 0.11 |
| PredHydroxy_1 | 0.02 | 0.99 | 0.64 | 0.50 | 0.03 | 0.03 |
| PredHydroxy_2 | 0.00 | 0.99 | 0.00 | 0.50 | 0.00 | -0.07 |
| PredHydroxy_3 | 0.00 | 1.00 | nan | 0.50 | 0.00 | nan |
| RF-Hydroxysite | 0.50 | 0.27 | 0.40 | 0.38 | 0.45 | -0.24 |
| RF-Hydroxysite_1 | 0.38 | 0.41 | 0.39 | 0.39 | 0.38 | -0.22 |
| RF-Hydroxysite_2 | 0.22 | 0.60 | 0.35 | 0.41 | 0.27 | -0.20 |
| ASM3 | 0.15 | 0.82 | 0.44 | 0.48 | 0.22 | -0.05 |
| Consensus | 0.02 | 0.99 | 0.66 | 0.50 | 0.04 | 0.04 |
| Random | 0.51 | 0.50 | 0.50 | 0.50 | 0.50 | 0.00 |
| Naïve-HMM | 0.87 | 0.97 | 0.97 | 0.92 | 0.91 | 0.84 |

**Table B. Performance on the MS-HeLa dataset.**

| **Method** | **Sn** | **Sp** | **Prec** | **Wacc** | **F1** | **Mcc** |
| --- | --- | --- | --- | --- | --- | --- |
| HydPred | 0.10 | 0.97 | 0.79 | 0.54 | 0.17 | 0.15 |
| ModPred | 0.32 | 0.78 | 0.60 | 0.55 | 0.42 | 0.12 |
| ModPred_1 | 0.22 | 0.88 | 0.65 | 0.55 | 0.33 | **0.13** |
| ModPred_2 | 0.04 | 0.99 | 0.81 | 0.52 | 0.08 | 0.10 |
| iHyd-PseAAC | 0.15 | 0.92 | 0.65 | 0.54 | 0.25 | 0.11 |
| OH-Pred | 0.17 | 0.90 | 0.63 | 0.54 | 0.27 | 0.11 |
| PredHydroxy | 0.07 | 0.98 | 0.77 | 0.53 | 0.13 | 0.12 |
| PredHydroxy_1 | 0.04 | 0.99 | 0.81 | 0.52 | 0.08 | 0.10 |
| PredHydroxy_2 | 0.03 | 0.99 | 0.76 | 0.51 | 0.05 | 0.07 |
| PredHydroxy_3 | 0.00 | 1.00 | 1.00 | 0.50 | 0.00 | 0.03 |
| RF-Hydroxysite | 0.64 | 0.27 | 0.47 | 0.46 | 0.54 | -0.10 |
| RF-Hydroxysite_1 | 0.53 | 0.41 | 0.47 | 0.47 | 0.50 | -0.06 |
| RF-Hydroxysite_2 | 0.35 | 0.60 | 0.46 | 0.47 | 0.40 | -0.06 |
| ASM3 | 0.21 | 0.82 | 0.53 | 0.51 | 0.30 | 0.03 |
| Consensus | 0.04 | 0.99 | 0.79 | 0.52 | 0.08 | 0.09 |
| Random | 0.49 | 0.50 | 0.50 | 0.50 | 0.49 | -0.01 |
| Naïve-HMM | 0.95 | 0.97 | 0.97 | 0.96 | 0.96 | 0.92 |

**Table C. Performance on the MS-Kim dataset.**

| **Method** | **Sn** | **Sp** | **Prec** | **Wacc** | **F1** | **Mcc** |
| --- | --- | --- | --- | --- | --- | --- |
| HydPred | 0.09 | 0.97 | 0.78 | 0.53 | 0.17 | 0.14 |
| ModPred | 0.35 | 0.78 | 0.62 | 0.57 | 0.45 | 0.15 |
| ModPred_1 | 0.24 | 0.88 | 0.67 | 0.56 | 0.36 | 0.16 |
| ModPred_2 | 0.05 | 0.99 | 0.82 | 0.52 | 0.09 | 0.11 |
| iHyd-PseAAC | 0.14 | 0.92 | 0.63 | 0.53 | 0.23 | 0.10 |
| OH-Pred | 0.19 | 0.90 | 0.65 | 0.54 | 0.29 | 0.12 |
| PredHydroxy | 0.07 | 0.98 | 0.76 | 0.52 | 0.13 | 0.12 |
| PredHydroxy_1 | 0.04 | 0.99 | 0.80 | 0.51 | 0.07 | 0.09 |
| PredHydroxy_2 | 0.02 | 0.99 | 0.72 | 0.51 | 0.04 | 0.05 |
| PredHydroxy_3 | 0.00 | 1.00 | 1.00 | 0.50 | 0.00 | 0.03 |
| RF-Hydroxysite | 0.62 | 0.27 | 0.46 | 0.44 | 0.53 | -0.12 |
| RF-Hydroxysite_1 | 0.50 | 0.41 | 0.46 | 0.46 | 0.48 | -0.09 |
| RF-Hydroxysite_2 | 0.33 | 0.60 | 0.45 | 0.46 | 0.38 | -0.08 |
| ASM3 | 0.20 | 0.82 | 0.52 | 0.51 | 0.29 | 0.02 |
| Consensus | 0.04 | 0.99 | 0.78 | 0.51 | 0.07 | 0.09 |
| Random | 0.50 | 0.50 | 0.50 | 0.50 | 0.50 | -0.01 |
| Naïve-HMM | 0.94 | 0.97 | 0.97 | 0.95 | 0.95 | 0.91 |

**Table D. Performance on the MS dataset.** The MS-Kim is merged with MS-HeLa.

| **Method** | **Sn** | **Sp** | **Prec** | **Wacc** | **F1** | **Mcc** |
| --- | --- | --- | --- | --- | --- | --- |
| HydPred | 0.80 | 0.97 | 0.97 | 0.89 | 0.88 | 0.79 |
| ModPred | 0.94 | 0.78 | 0.81 | 0.86 | 0.87 | 0.73 |
| ModPred_1 | 0.91 | 0.88 | 0.89 | 0.90 | 0.90 | 0.79 |
| ModPred_2 | 0.47 | 0.99 | 0.98 | 0.73 | 0.63 | 0.54 |
| iHyd-PseAAC | 0.80 | 0.92 | 0.91 | 0.86 | 0.85 | 0.73 |
| OH-Pred | 0.98 | 0.90 | 0.91 | 0.94 | 0.94 | 0.88 |
| PredHydroxy | 0.73 | 0.98 | 0.97 | 0.85 | 0.83 | 0.73 |
| PredHydroxy_1 | 0.64 | 0.99 | 0.99 | 0.81 | 0.77 | 0.67 |
| PredHydroxy_2 | 0.49 | 0.99 | 0.98 | 0.74 | 0.66 | 0.56 |
| PredHydroxy_3 | 0.00 | 1.00 | nan | 0.50 | 0.00 | nan |
| RF-Hydroxysite | 0.03 | 0.27 | 0.03 | 0.15 | 0.03 | -0.73 |
| RF-Hydroxysite_1 | 0.03 | 0.41 | 0.04 | 0.22 | 0.03 | -0.61 |
| RF-Hydroxysite_2 | 0.01 | 0.60 | 0.03 | 0.31 | 0.02 | -0.48 |
| ASM3 | 0.13 | 0.82 | 0.41 | 0.47 | 0.19 | -0.08 |
| Consensus | 0.64 | 0.99 | 0.98 | 0.81 | 0.77 | 0.67 |
| Random | 0.49 | 0.50 | 0.49 | 0.49 | 0.49 | -0.01 |
| Naïve-HMM | 0.87 | 0.97 | 0.97 | 0.92 | 0.91 | 0.84 |

**Table E. Performance on the Literature-collagen dataset.**

| **Method** | **Sn** | **Sp** | **Prec** | **Wacc** | **F1** | **Mcc** |
| --- | --- | --- | --- | --- | --- | --- |
| HydPred | 0.23 | 0.97 | 0.90 | 0.60 | 0.37 | 0.30 |
| ModPred | 0.85 | 0.78 | 0.80 | 0.82 | 0.82 | 0.64 |
| ModPred_1 | 0.77 | 0.88 | 0.87 | 0.82 | 0.81 | 0.65 |
| ModPred_2 | 0.22 | 0.99 | 0.96 | 0.61 | 0.36 | 0.33 |
| iHyd-PseAAC | 0.44 | 0.92 | 0.85 | 0.68 | 0.58 | 0.41 |
| OH-Pred | 0.69 | 0.90 | 0.87 | 0.79 | 0.77 | 0.60 |
| PredHydroxy | 0.41 | 0.98 | 0.95 | 0.69 | 0.57 | 0.47 |
| PredHydroxy_1 | 0.26 | 0.99 | 0.96 | 0.63 | 0.41 | 0.37 |
| PredHydroxy_2 | 0.10 | 0.99 | 0.93 | 0.55 | 0.19 | 0.21 |
| PredHydroxy_3 | 0.01 | 1.00 | 1.00 | 0.51 | 0.02 | 0.09 |
| RF-Hydroxysite | 0.19 | 0.27 | 0.21 | 0.23 | 0.20 | -0.55 |
| RF-Hydroxysite_1 | 0.14 | 0.41 | 0.19 | 0.27 | 0.16 | -0.47 |
| RF-Hydroxysite_2 | 0.08 | 0.60 | 0.17 | 0.34 | 0.11 | -0.37 |
| ASM3 | 0.08 | 0.82 | 0.32 | 0.45 | 0.13 | -0.14 |
| Consensus | 0.17 | 0.99 | 0.94 | 0.58 | 0.28 | 0.27 |
| Random | 0.49 | 0.50 | 0.49 | 0.49 | 0.49 | -0.02 |
| Naïve-HMM | 0.75 | 0.97 | 0.96 | 0.86 | 0.84 | 0.73 |

**Table F. Performance on the MS-collagen dataset.**


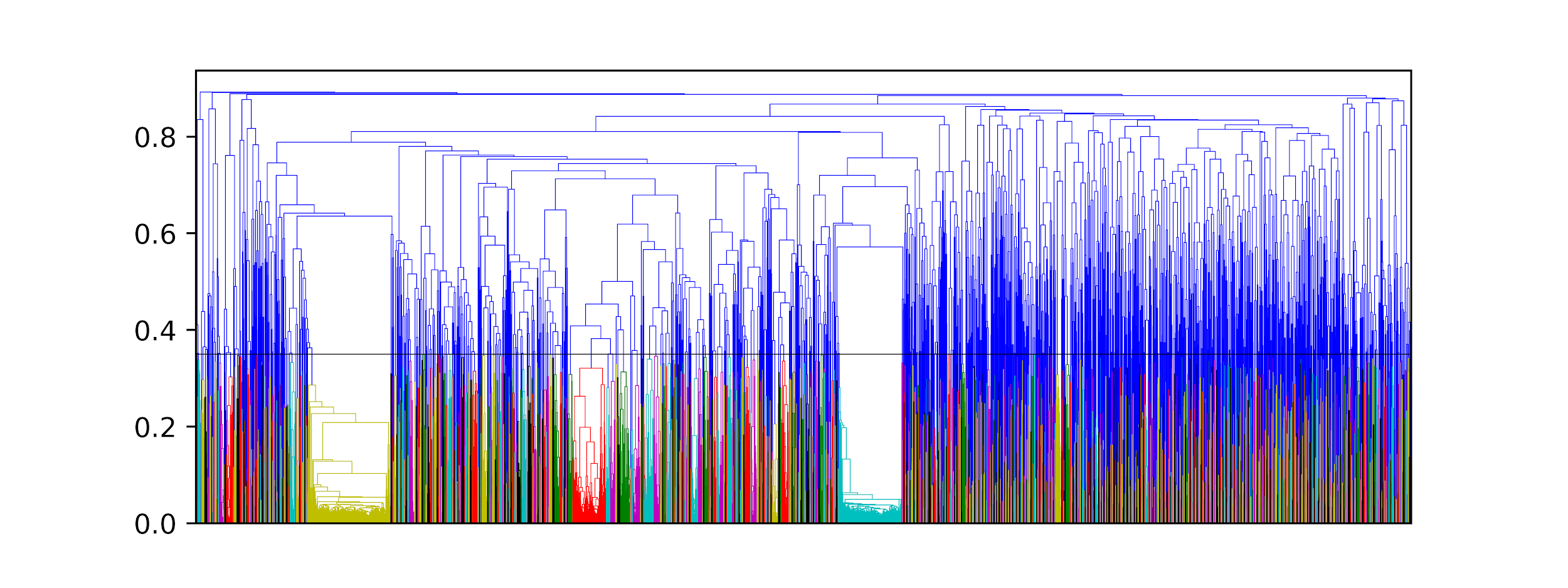


**Figure A. Dendrogram of hydroxylated and non hydroxylated sites.** Nodes of the same color have a UPGMA distance closer than 0.35. The distance is calculated as the inverse of the sum of the Blosum62 score for each pair of residues with a penalty of -5 and -1 for gap opening and extension respectively (gaps are only possible for sites shorter than the window, i.e. close to the sequence end). The three larger groups (yellow, red, ciano) correspond to three different collagen motifs.


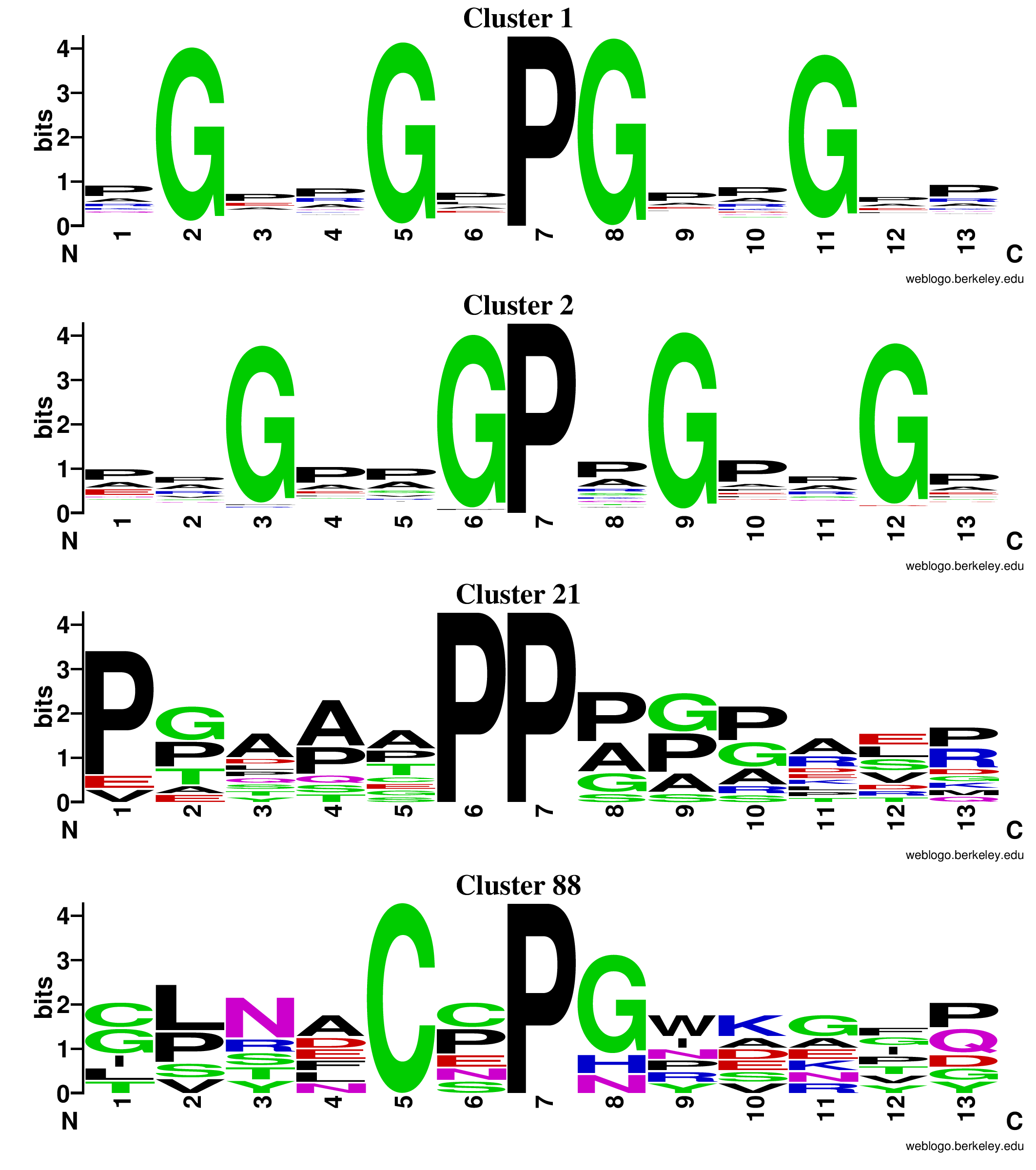


**Figure B. Sequence logo of collagen clusters**

The fifth logo mentioned in the manuscript is missing as the corresponding cluster, #559, contains only one site sequence, “CSFECQPARGPPG”.


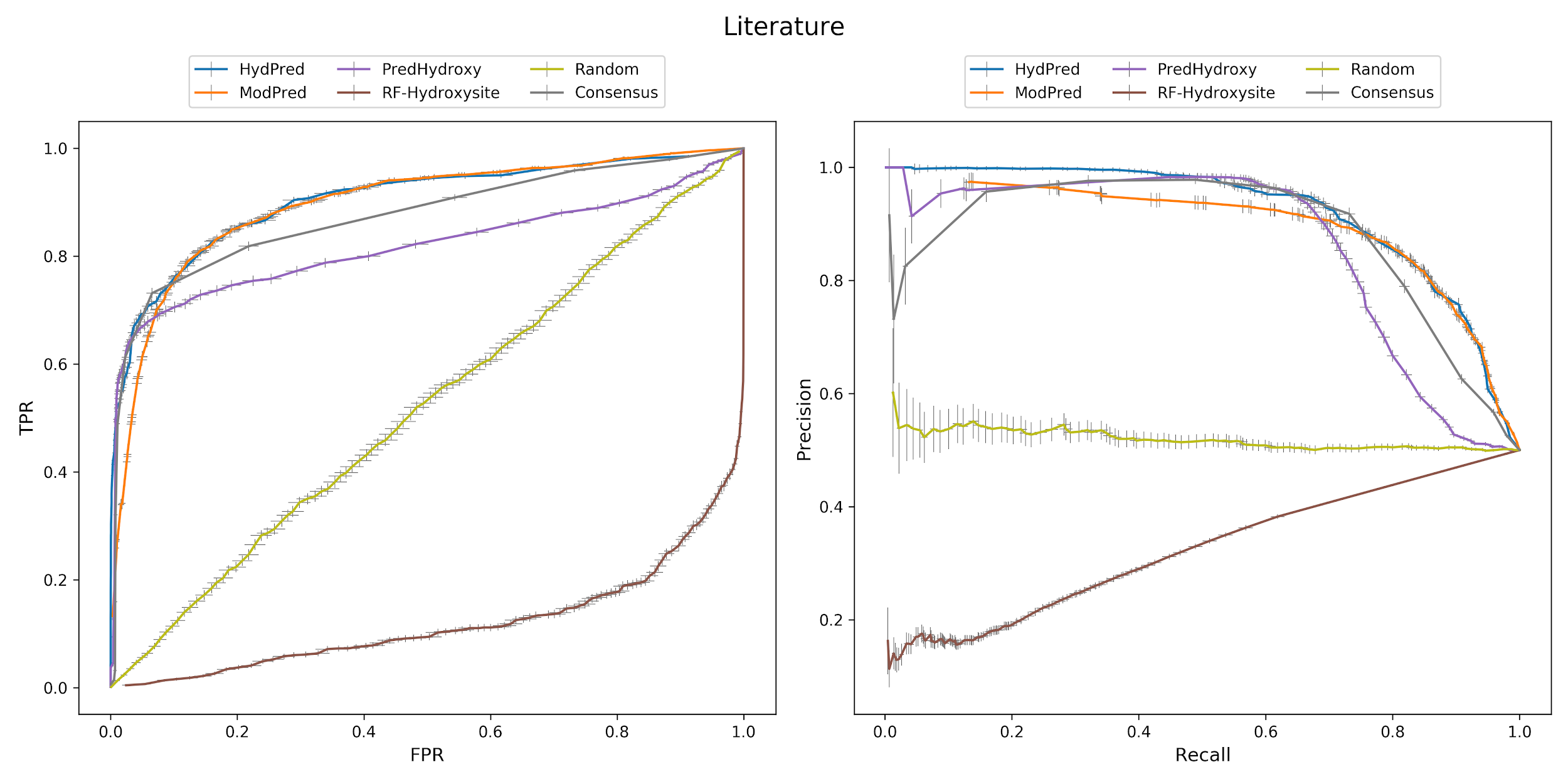


**Figure C. ROC and precision-recall curves on the Literature dataset.**

**
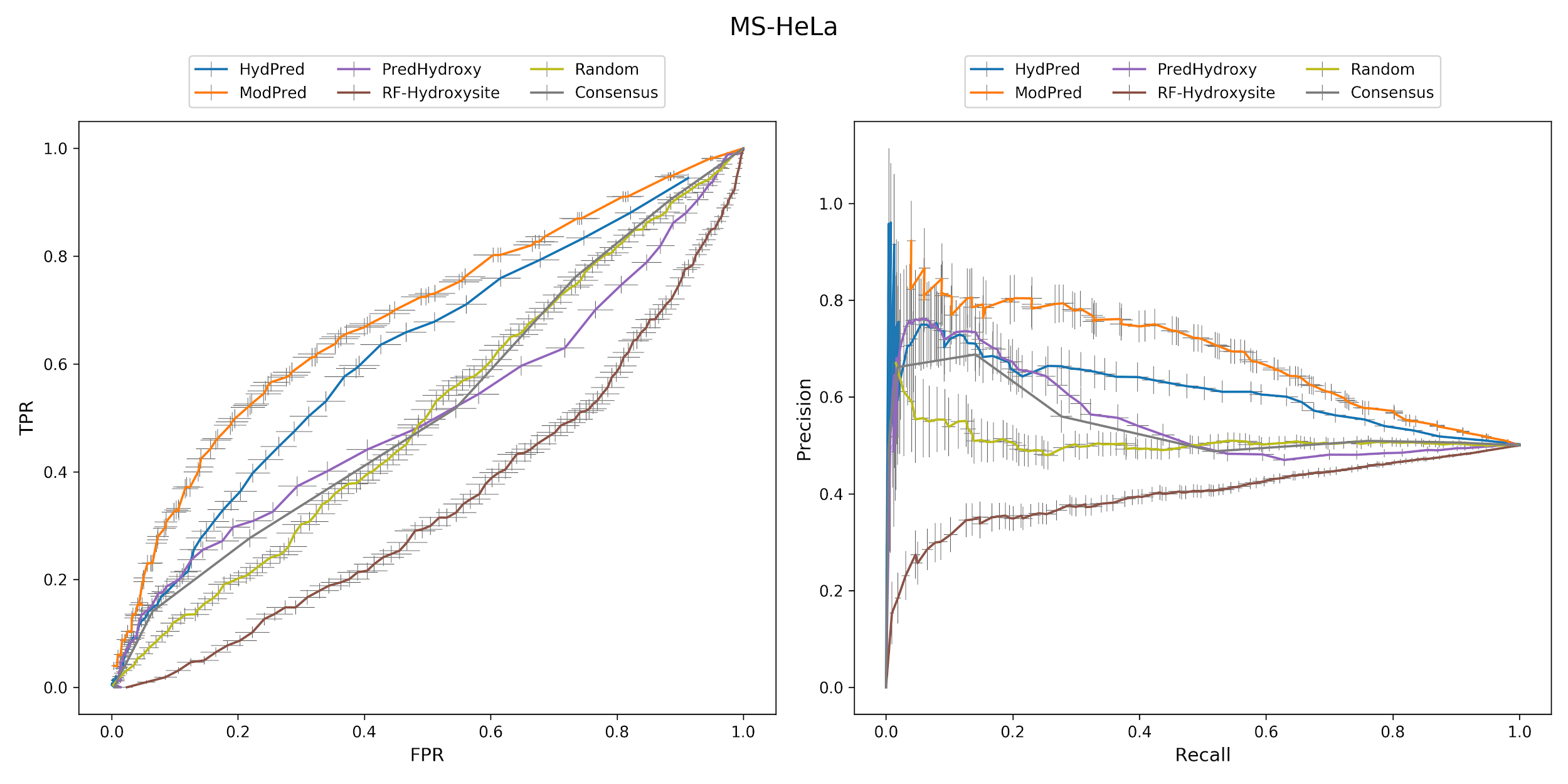
**

**Figure D. ROC and precision-recall curves on the MS-HeLa dataset.**


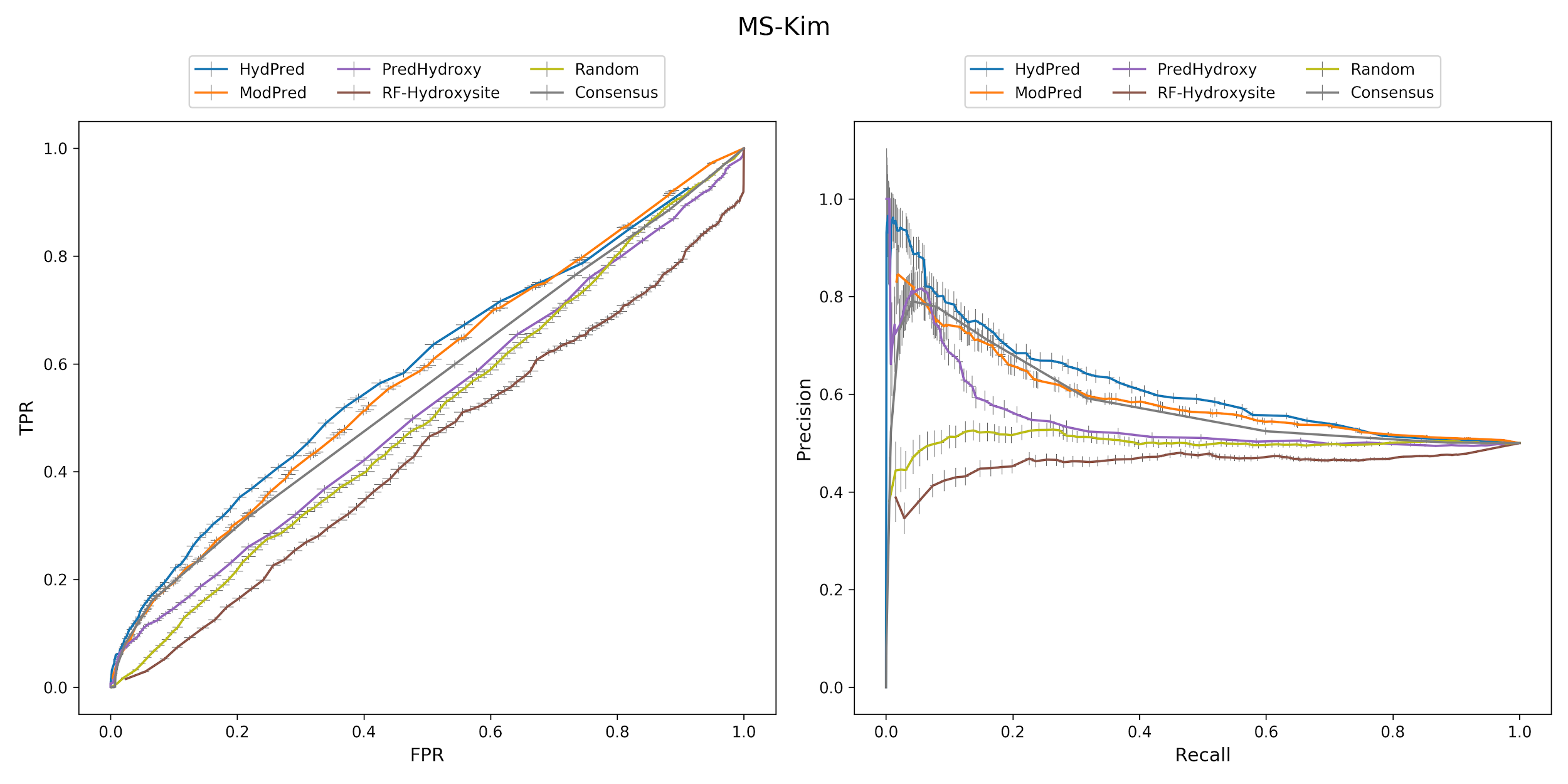


**Figure E. ROC and precision-recall curves on the MS-Kim dataset.**

**
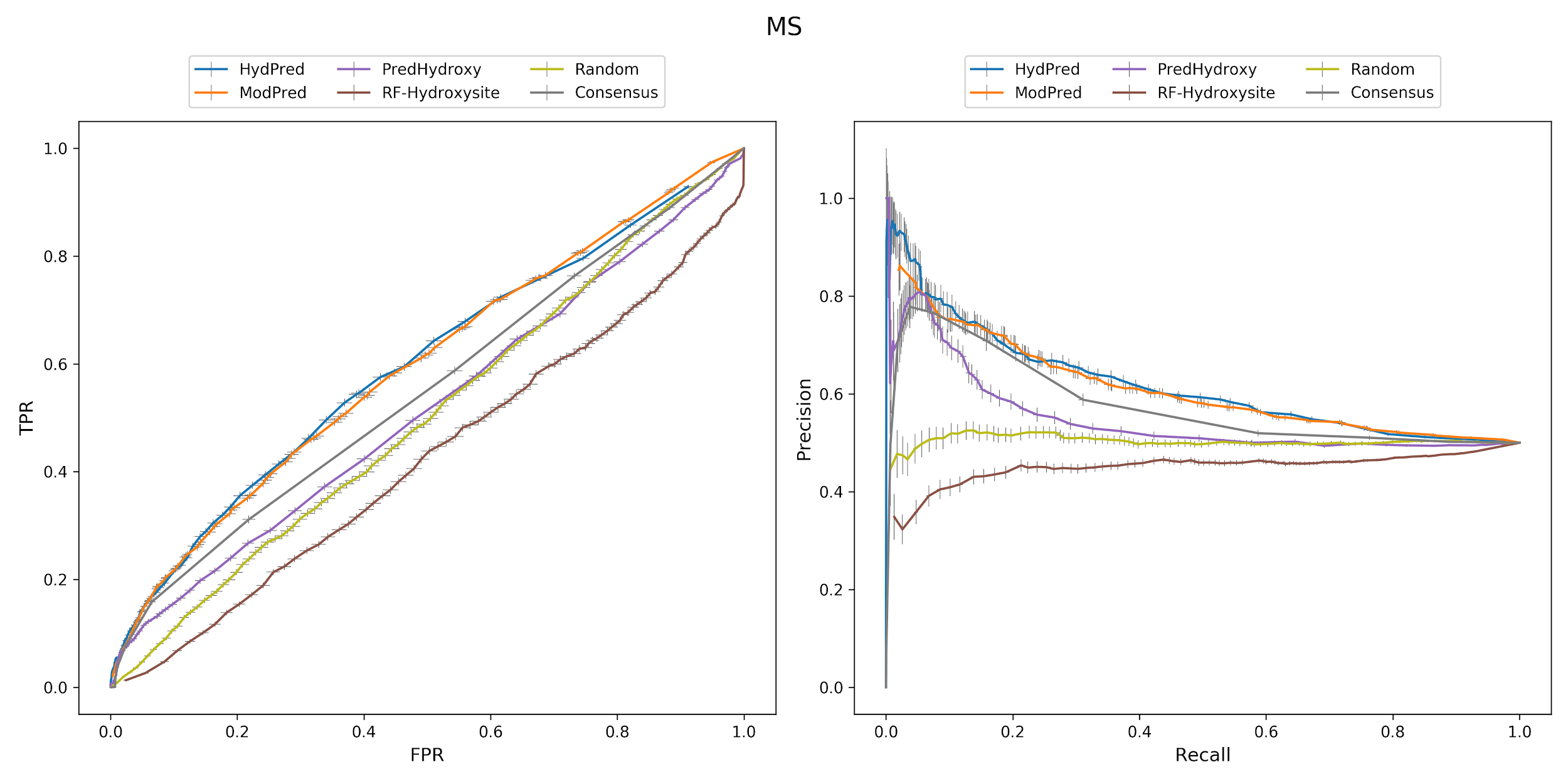
**

**Figure F. ROC and precision-recall curves on the MS dataset (MS-Kim merged with MS-HeLa).**

**
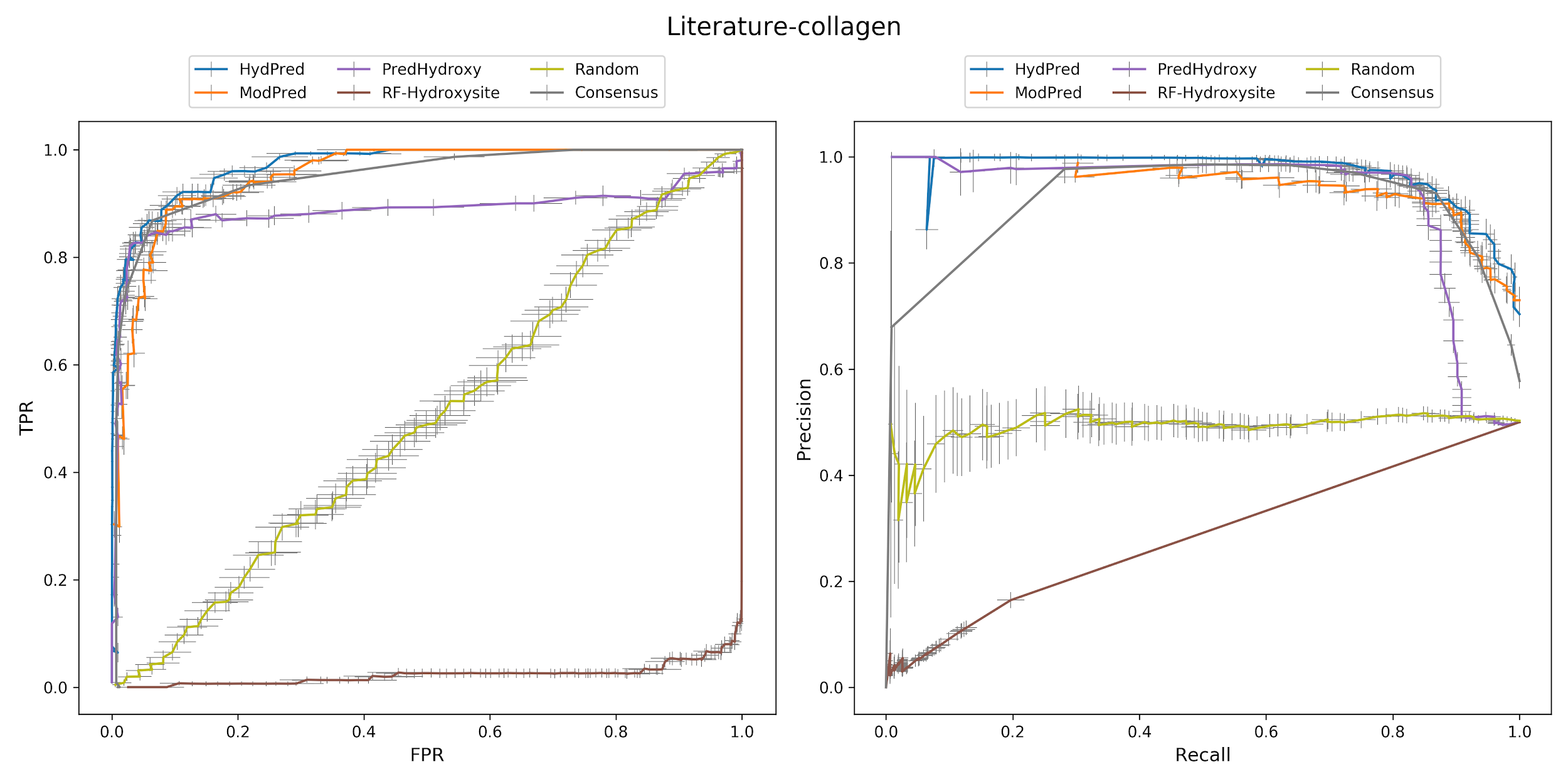
**

**Figure G. ROC and precision-recall curves on the Literature-collagen dataset.**

**
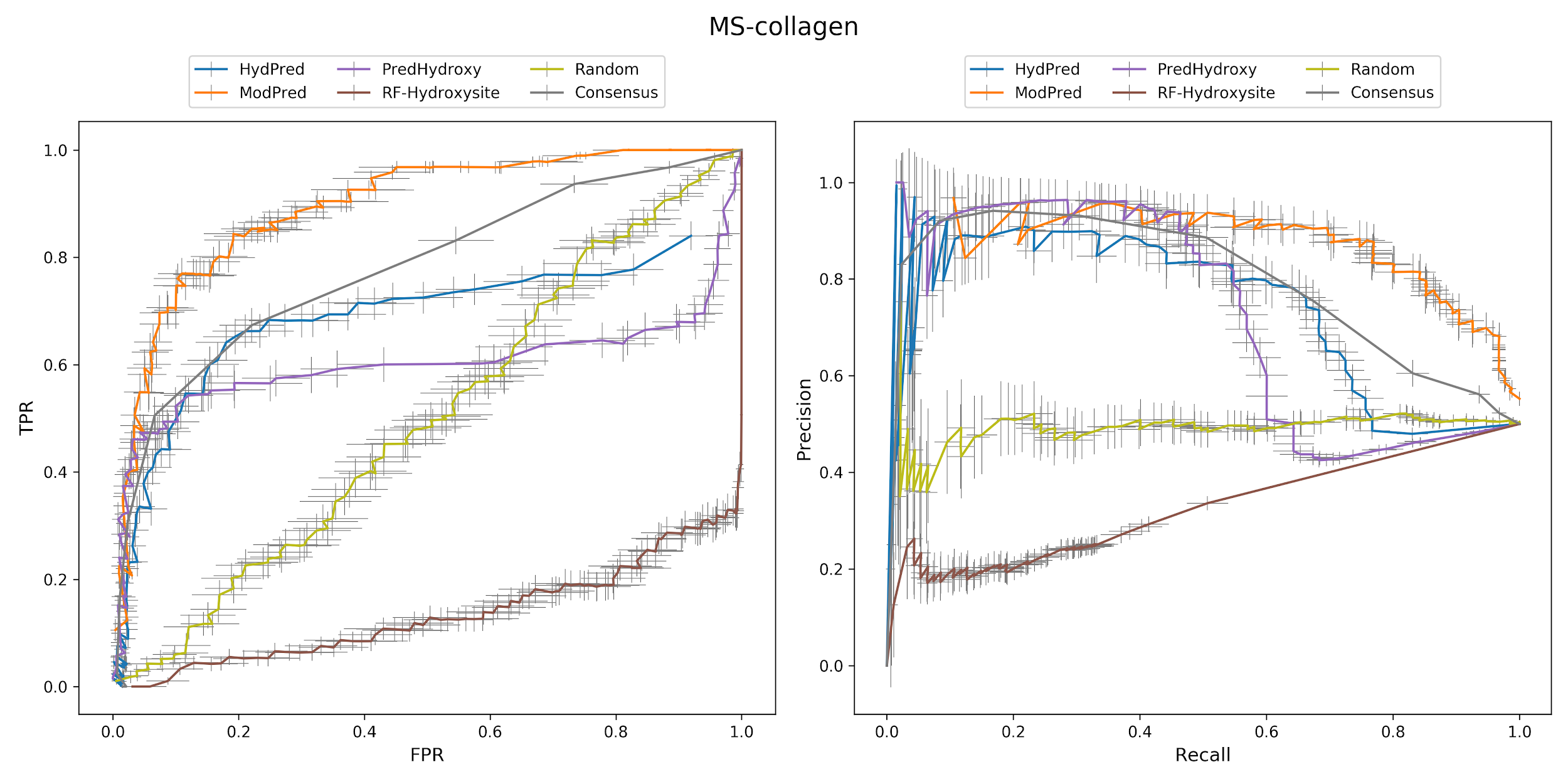
**

**Figure H. ROC and precision-recall curves on the MS-collagen dataset.**
